# The impact of COVID-19 on pulmonary, neurological, and cardiac outcomes: evidence from a Mendelian randomization study

**DOI:** 10.3389/fpubh.2023.1303183

**Published:** 2023-12-14

**Authors:** Pooja U. Shenoy, Hrushikesh Udupa, Jyothika KS, Sangeetha Babu, Nikshita K, Neha Jain, Ranajit Das, Priyanka Upadhyai

**Affiliations:** ^1^Division of Data Analytics, Bioinformatics and Structural Biology, Yenepoya Research Centre, Yenepoya (Deemed to be University), Mangalore, India; ^2^Department of Community Medicine, Yenepoya Medical College and Hospital, Yenepoya (Deemed to be University), Mangalore, India; ^3^Department of Statistics, Yenepoya (Deemed to be University), Mangalore, India; ^4^Department of Medical Genetics, Kasturba Medical College, Manipal, Manipal Academy of Higher Education, Manipal, India

**Keywords:** long COVID, Mendelian randomization, COVID-induced neurological disorders, COVID-induced fatigue, COVID-induced cardiac disease

## Abstract

**Background:**

Long COVID is a clinical entity characterized by persistent health problems or development of new diseases, without an alternative diagnosis, following SARS-CoV-2 infection that affects a significant proportion of individuals globally. It can manifest with a wide range of symptoms due to dysfunction of multiple organ systems including but not limited to cardiovascular, hematologic, neurological, gastrointestinal, and renal organs, revealed by observational studies. However, a causal association between the genetic predisposition to COVID-19 and many post-infective abnormalities in long COVID remain unclear.

**Methods:**

Here we employed Mendelian randomization (MR), a robust genetic epidemiological approach, to investigate the potential causal associations between genetic predisposition to COVID-19 and long COVID symptoms, namely pulmonary (pneumonia and airway infections including bronchitis, emphysema, asthma, and rhinitis), neurological (headache, depression, and Parkinson’s disease), cardiac (heart failure and chest pain) diseases, and chronic fatigue. Using two-sample MR, we leveraged genetic data from a large COVID-19 genome-wide association study and various disorder-specific datasets.

**Results:**

This analysis revealed that a genetic predisposition to COVID-19 was significantly causally linked to an increased risk of developing pneumonia, airway infections, headache, and heart failure. It also showed a strong positive correlation with chronic fatigue, a frequently observed symptom in long COVID patients. However, our findings on Parkinson’s disease, depression, and chest pain were inconclusive.

**Conclusion:**

Overall, these findings provide valuable insights into the genetic underpinnings of long COVID and its diverse range of symptoms. Understanding these causal associations may aid in better management and treatment of long COVID patients, thereby alleviating the substantial burden it poses on global health and socioeconomic systems.

## 1 Introduction

Long-term residual health problems or the onset of new diseases following Coronavirus disease 2019 (COVID-19) that is caused by severe acute respiratory syndrome coronavirus 2 (SARS-CoV-2) are termed as post-acute sequalae of SARS-CoV-2 (PASC) or Long COVID that is a recognized and multi-systemic clinical entity with onset ≥3 months after a probable or confirmed diagnosis of COVID-19 and lasting for ≥2 months, with symptoms not explained by any other alternative diagnosis (1). It can manifest with a broad range of clinical phenotypes owing to respiratory, cardiovascular, neurological, gastrointestinal, renal, immunological, reproductive and other organ system dysfunction (2). Its global prevalence varies widely, ranging from 7.5–45% individuals including children and adolescents (3–7) and even conservatively it is estimated to affect ~65 million individuals globally (2); though it is likely this number is much higher due to the large number of unreported cases. The etiology of long COVID is complex and influenced by several factors, such as severity of initial infection, age, sex, presence of comorbidities, e.g., obesity and host genetic variations, such as at the *FOXP4* locus (8–13). The burden of long COVID was also found to be significant in individuals with SARS-CoV-2 breakthrough infections (BTI) that occur post receiving vaccine booster dose (14). However, vaccination partially ameliorated the risk of mortality and extreme adverse outcomes associated with BTI in long COVID subjects (14, 15).

Frequently observed long COVID symptoms include chronic fatigue and respiratory abnormalities, e.g., dyspnea or shortness of breath (5, 11, 16). Dyspnea and cough were found to persist in 40 and 20% long COVID subjects, respectively (17). Follow ups in individuals hospitalized with COVID-19 induced pneumonia, using radiological and pulmonary function investigations, uncovered attenuated lung function, fibrotic-like changes in the lungs and parenchymal lung disease in long COVID patients (18–20). Cognitive and neurological derangements are another major cluster of long COVID symptoms, including anosmia, ageusia, headache, cognitive impairments, e.g., memory and attention deficits, brain fog, neuropsychiatric symptoms, such as depression, anxiety, psychosis, and insomnia (16, 21–25). Few cases of parkinsonism have also been reported in the older long COVID patients (26, 27). In addition, observational studies note that the cardiovascular sequalae of long COVID include chest pain, heart failure, dysrhythmias, cerebrovascular disorders, inflammatory heart disease, ischemic heart disease and thromboembolic disease (28, 29). Further the adverse health outcomes in long COVID include the new onset of diseases, such as type 2 diabetes (30) and multi-organ damage (31). Given that a plethora of long COVID manifestations is not only observed in acute cases of COVID-19 but also significantly impact individuals with asymptomatic, mild or moderate initial SARS-CoV-2 infections that did not warrant hospitalization (7, 32, 33), severely exacerbates the health and socio-economic burden of long COVID worldwide.

The causal association between the genetic predisposition to COVID-19 and many post-infective abnormalities in long COVID remain unclear. Mendelian randomization (MR) is a genetic epidemiological strategy that utilizes genetic variants, such as single nucleotide polymorphisms (SNPs) associated with an exposure as instrumental variables to determine the causal relationship between the exposure and health outcome (34). It is less susceptible to confounding, reverse causality and regression dilution biases that limit observational studies. So far MR studies have been used to dissect a causal association of COVID-19 with the increased risk of development of Alzheimer’s disease (35), hypothyroidism (36), psychosis and schizophrenia (37), and specific cancers (38).

In the present study we used two sample MR to investigate the potential causal association of genetic predisposition to COVID-19 (exposure) with the onset of long COVID symptoms (outcomes), namely pulmonary disease (airway infections and pneumonia), neurological deficits (headache, depression, and parkinsonism), cardiac anomalies (chest pain and heart failure) and chronic fatigue.

## 2 Methods

### 2.1 Study design

**Figure 1:**
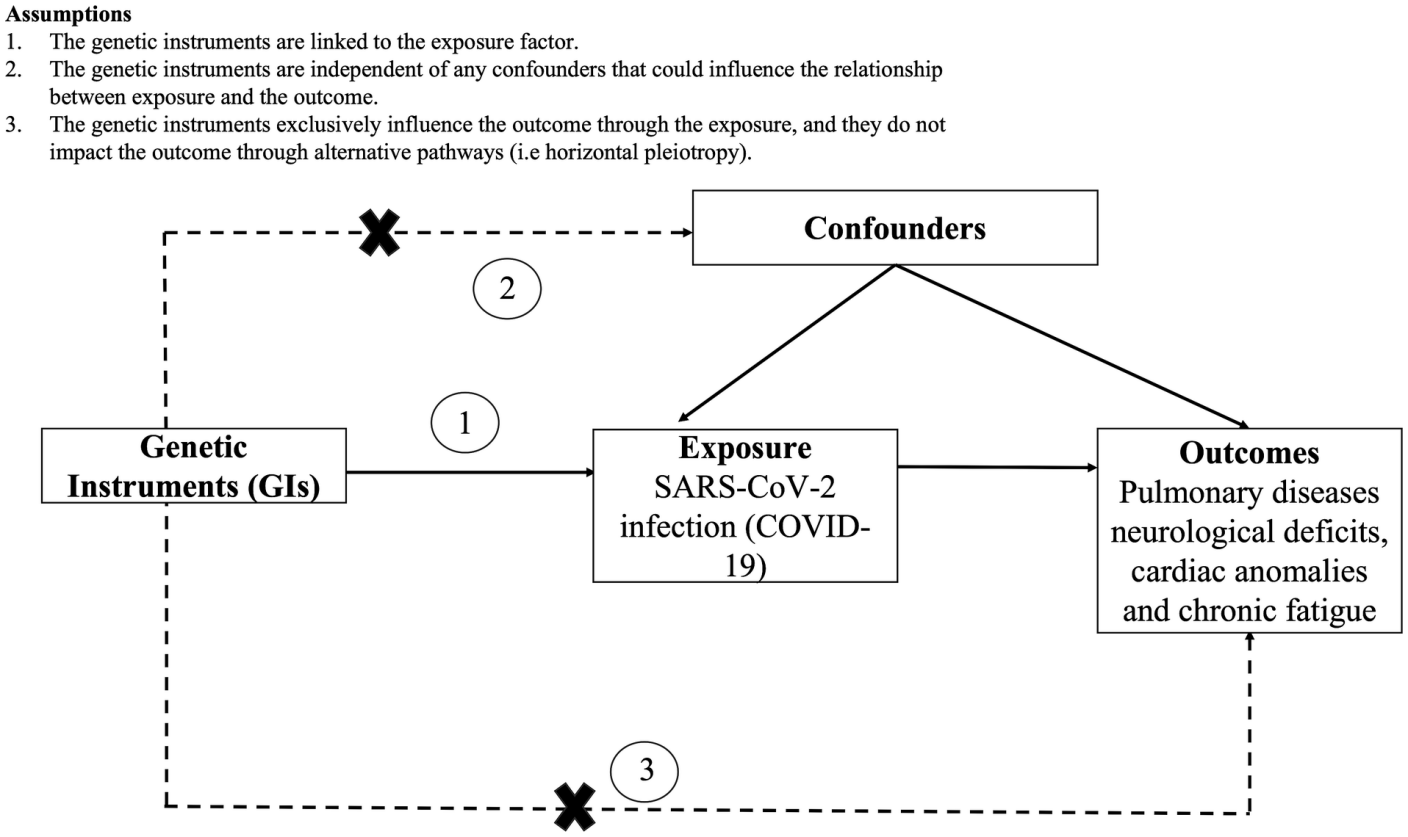
Study design and assumptions of Mendelian randomization (MR) analysis along with the assumptions.

Mendelian randomization (MR) relies on genetic variations as tools to investigate the lifelong and causal impacts of an exposure on an outcome (39). It closely resembles randomized controlled trials, as alleles are randomly assigned at conception, reducing susceptibility to reverse causation and unaccounted-for variables compared to traditional cohort studies. Consequently, it offers more robust evidence for establishing causal relationships. In the current study, we employed a two-sample MR approach by extracting exposure and outcome summary data from separate publicly available datasets. Notably, the effectiveness and reliability of these findings hinge on three key assumptions: (a) genetic instruments are linked to the exposure; (b) genetic instruments are not associated with any confounding factors that influence the exposure-outcome connection; (c) genetic instruments solely impact the outcome through the exposure, without involving other pathways (39). To statistically evaluate these assumptions, we performed pleiotropy and heterogeneity tests. The study design and underlying assumptions for MR is shown in Figure 1.

### 2.2 Data source: COVID-19

Genome Wide Association Study (GWAS) summary statistics were retrieved from one of the largest GWAS data set for COVID-19 (hospitalized vs. non-hospitalized) described by the COVID-19 Host Genetics Initiative Release 6: *B1_ALL_leave_23andme,* released on June 15, 2021. *B1_ALL_leave_23andme* consists of trans-ancestry GWAS summary statistics of individuals who were hospitalized (*n* = 14,480; cases) vs. those who were not hospitalized (*n* = 73,191; controls) due to COVID-19 (Table 1). 1,35,110 highly significant SNPs were identified (*p* ≤ 0.01). To ensure the independence of these SNPs, they were first extracted from the Genome Asia dataset (40) and subsequently pruned for linkage disequilibrium (LD) using PLINK v1.9 (41). The LD proxies were limited to a minimum *r*^2^ ≧ 0.6. 1,02,354 SNPs were pruned out and the remaining 32,756 SNPs were used for downstream analyses.

**Table 1:** Various disorders and COVID-19 genetic summary data sources.

Trait	Sample size	No. of cases	No. of controls	No. of SNPs assessed	Population
COVID-19	87,671	14,480	73,191	–	Trans-ancestry
Pneumonia	486,484	22,567	463,917	12,243,546	European
Bronchitis, emphysema, asthma, rhinitis	8,562	5,986	2,576	9,809,165	South Asian
Anxiety, tension, depression	8,438	828	7,610	9,809,832	South Asian
Parkinson’s disease	482,730	33,674	449,056	17,891,936	European
Headache	8,820	348	8,472	9,801,707	South Asian
Heart failure	977,323	47,309	930,014	7,773,021	European
Chest pain	8,788	1,267	7,521	9,810,370	South Asian
Fatigue (tiredness/lethargy)	7,878	7,878	Population control	9,811,636	South Asian

### 2.3 Data source: disorders

Information about genetic association of pulmonary diseases (airway infections and pneumonia), neurological deficits (headache, depression and Parkinson’s Disease), cardiac anomalies (chest pain and heart failure) and chronic fatigue were obtained from the *ieu open gwas project*^1^ (Table 1). The pneumonia dataset (ieu-b-4976) consisted of individuals with pneumonia (*n* = 22,567; cases) and individuals without pneumonia (*n* = 4,63,917; controls) of European ancestry. The dataset for a group of diseases that cause airflow blockage and breathing-related problems including bronchitis, emphysema, asthma, and rhinitis (ukb-e-6152_p1_CSA) consisted of 5,986 cases and 2,576 controls of South Asian ancestry. The GWAS summary statistics of anxiety, tension, depression (ukb-e-2100_CSA) consisted of individuals with depression (*n* = 828; cases) and individuals without depression (*n* = 7,610; controls) of South Asian ancestry. The Parkinson’s disease dataset (ieu-b-7) consisted of individuals with Parkinson’s disease (*n* = 33,674; cases) and individuals without Parkinson’s disease (*n* = 4,49,056; controls) of European ancestry. The headache dataset (ukb-e-339_CSA) consisted of individuals with headache (*n* = 348; cases) and individuals without headache (*n* = 8,472; controls) of South Asian ancestry. The summary statistics of heart failure (ebi-a-GCST009541) consisted of individuals with heart failure (*n* = 47,309; cases) and individuals without heart failure (*n* = 930,014; controls) of European ancestry. The chest pain dataset (ukb-e-418_CSA) consisted of individuals with chest pain (*n* = 1,267; cases) and individuals without chest pain (*n* = 7,521; controls) of South Asian ancestry. Notably, this dataset consists of patients with “nonspecific” chest pain, which indicates that the cause of the pain was unclear and may be caused by heart or lung abnormalities. Finally, the fatigue (tiredness/lethargy) dataset (ukb-e-2080_CSA) consisted of individuals with tiredness (*n* = 7,878; cases) of South Asian ancestry (Table 1).

### 2.4 Pleiotropy test

The intercept from MR-Egger regression (variants uncorrelated, random-effect model) implemented in the R package MendelianRandomization v0.9 (42), was utilized to test the pleiotropy of the SNPs associated with COVID-19 in pulmonary diseases (airway infections and pneumonia), neurological deficits (headache, depression and Parkinsonism), cardiac anomalies (chest pain and heart failure) and chronic fatigue (Table 2). In particular, MR assumes no pleiotropy, so a *p* > 0.05 implies no significant pleiotropy of the COVID-19 associated SNPs in above mentioned outcomes. Sensitivity analysis utilizing the “leave-one-out” method was performed using the mr_loo function in MendelianRandomization to evaluate whether the analysis could be influenced by a solitary SNP with significant and broad horizontal pleiotropic impact. In addition, funnel plots were generated using the mr_funnel function, were used to evaluate diversity among different SNPs.

**Table 2:** Pleiotropy test of COVID-19 associated SNPs in the diseases’ Genome Wide Association Study (GWAS).

Outcomes	No. of SNPs	mr_egger intercept	SE	*p*-value	*I*^2^*
Pneumonia	202	0.001	0.004	0.799	0.00%
Bronchitis, emphysema, asthma, rhinitis	274	−0.004	0.021	0.864	0.00%
Anxiety, tension, depression	235	0.032	0.031	0.295	0.00%
Parkinson’s disease	222	0.018	0.011	0.085	0.00%
Headache	219	−0.023	0.05	0.642	0.00%
Heart failure	212	0.006	0.005	0.179	0.00%
Chest pain	231	0.044	0.027	0.104	0.00%
Fatigue (tiredness/lethargy)	342	−0.01	0.01	0.311	0.00%

^*^I^2^ = 0.0% indicates no significant heterogeneity in the COVID-19 associated SNPs in the above-mentioned diseases GWAS.

### 2.5 Mendelian randomization analysis

To evaluate the causal association between COVID-19 and various pulmonary, neurological, and cardiac disorders and chronic fatigue, we performed MR analysis using *inverse variance weighted* (*IVW*), *Penalized IVW, Robust-IVW, Penalized robust IVW, Penalized MR-Egger, Robust MR-Egger, Mr*-*Egger*, *Penalized robust MR-Egger*, *weighted median, simple median* and *Penalized weighted median* estimators implemented in the R package MendelianRandomization v0.9. *p* value < 0.05 represents a causal association of COVID-19 with various disorders.

### 2.6 Single SNP effect analysis

The *mr_plot* function in MendelianRandomization v0.9 was used to visualize the individual potential causal effects of COVID-19 associated SNPs on various pulmonary, neurological, and cardiac disorders and chronic fatigue. Furthermore*, the mr_forest* function was used to determine single SNP effect size for COVID-19 on various disorders.

## 3 Results

### 3.1 Pleiotropy test

In terms of pleiotropy and sensitivity, the MR-Egger regression analysis demonstrated no indications of biased pleiotropy for any of the disorders under study (Table 2). The leave-one-out analysis and funnel plots also indicated no breaches of the instrumental variable assumptions (Supplementary Figure S1, respectively). The leave-one-out sensitivity analysis depicted that removing a specific SNP among COVID-19 SNPs did not change the results for any of the disease under study (Supplementary Figure S1).

**Figure 2:**
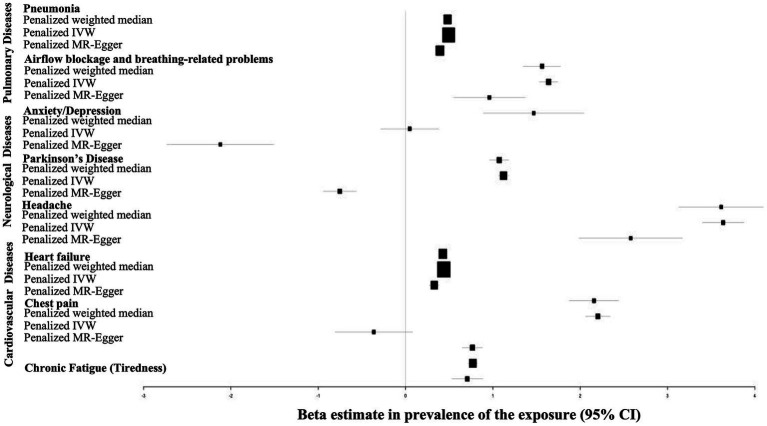
Forrest plot of MR analysis evaluating causal effects of genetic liability to COVID-19 on various pulmonary, cardiovascular and nueropsychiatric disorders. The plot was generated using the R package *forestplot* . The x-axis shows MR effect size for COVID-19 on various disorders. The y-axis shows the analysis for all SNPs together in a single instrument with the penalized IVW, penalized MR-Egger and penalized weighted median methods.

The overall Mendelian Randomization analysis results are summarized in Figure 2. Only penalized methods (penalized weighted median, penalized IVW and penalized MR-Egger) are shown. The plot was generated using the R package “forestplot.”

### 3.2 Effect of COVID-19 on lung function

**Figure 3:**
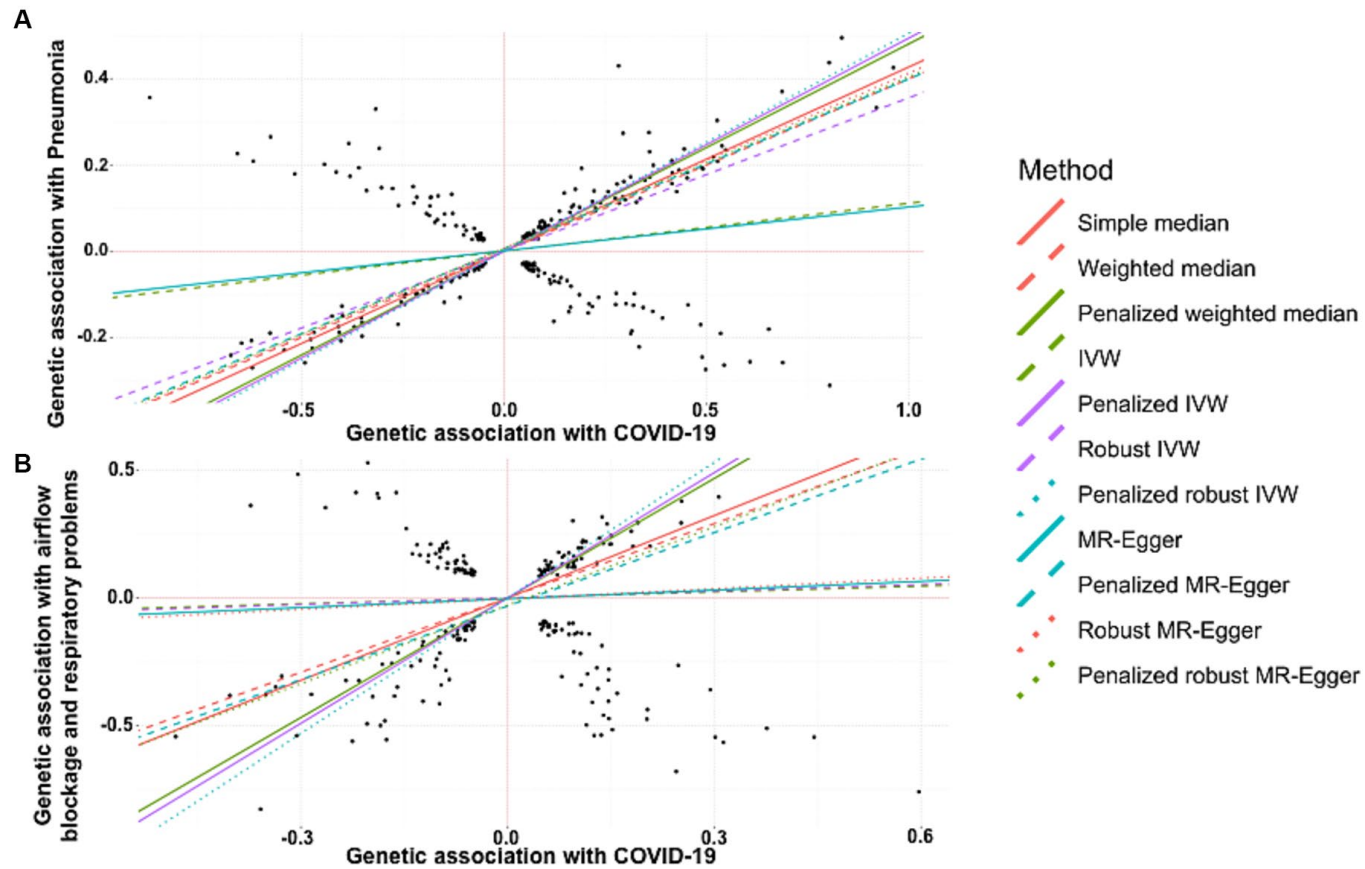
Scatter plot of SNPs associated with COVID-19 and pulmonary diseases. **(A)** pneumonia and **(B)** a group of diseases associated with airflow blockage and breathing-related problems including bronchitis, emphysema, asthma and rhinitis. The slopes of each line represent the causal association for each method. The plot was generated using the R package MendelianRandomization v0.9.

A strong positive association between history of COVID-19 and development of pneumonia was discerned by simple median analysis (β: 0.427, 95% CI 0.391 to 0.463, *p* < 0.0001), weighted median (β: −0.400, 95% CI 0.362 to 0.438, *p* < 0.0001), penalized weighted median (β: 0.481, 95% CI 0.437 to 0.526, *p* < 0.0001), IVW (β: 0.111, 95% CI 0.059 to 0.63, *p* < 0.0001), penalized IVW (β: 0.494, 95% CI 0.471 to 0.518, *p* < 0.0001), robust IVW (β: 0.356, 95% CI −0.018 to 0.730, *p* = 0.062), penalized robust IVW (β: 0.505, 95% CI 0.404 to 0.607, *p* < 0.0001), MR-Egger (β: 0.102, 95% CI 0.014 to 0.190, *p* = 0.023), penalized MR-Egger (β: 0.394, 95% CI 0.353 to 0.434, *p* < 0.0001), robust MR-Egger (β: 0.406, 95% CI 0.315 to 0.497, *p* < 0.0001) and penalized robust MR-Egger (β: 0.398, 95% CI 0.374 to 0.422, *p* < 0.0001) methods (Table 3A, Figure 3A).

**Table 3A:** The MR estimates of the causal effect of COVID-19 on pneumonia.

Method	Estimate	SE	95% CI		*p*-value
Simple median	0.427	0.018	0.391	0.463	<0.0001
Weighted median	0.400	0.019	0.362	0.438	<0.0001
Penalized weighted median	0.481	0.023	0.437	0.526	<0.0001
IVW	0.111	0.027	0.059	0.163	<0.0001
Penalized IVW	0.494	0.012	0.471	0.518	<0.0001
Robust IVW	0.356	0.191	−0.018	0.730	0.062
Penalized robust IVW	0.505	0.052	0.404	0.607	<0.0001
MR-Egger	0.102	0.045	0.014	0.190	0.023
Penalized MR-Egger	0.394	0.021	0.353	0.434	<0.0001
Robust MR-Egger	0.406	0.046	0.315	0.497	<0.0001
Penalized robust MR-Egger	0.398	0.012	0.374	0.422	<0.0001

MR revealed a positive and causal association between COVID-19 and onset of the group of diseases associated with airflow blockage and respiratory problems including bronchitis, emphysema, asthma, and rhinitis. This was evidenced by the simple median estimate (β: 1.077, 95% CI 0.862 to 1.292, *p* < 0.0001), weighted median (β: 0.971, 95% CI 0.751 to 1.191, *p* < 0.0001), penalized weighted median (β: 1.563, 95% CI 1.350 to 1.776, *p* < 0.0001), penalized IVW (β: 1.638, 95% CI 1.533 to 1.743, *p* < 0.0001), penalized robust IVW (β: 1.771, 95% CI 1.488 to 2.055, *p* < 0.0001), penalized MR-Egger (β: 0.960, 95% CI 0.550 to 1.371, *p* < 0.0001), and the penalized robust MR-Egger (β: 1.025, 95% CI 0.667 to 1.382, *p* < 0.0001) methods. While the estimates from IVW (β: 0.077, 95% CI −0.145 to 0.298, *p* = 0.497), robust IVW (β: 0.088, 95% CI −0.171 to 0.347, *p* = 0.505), MR-Egger (β: 0.115, 95% CI −0.371 to 0.600, *p* = 0.644) and robust MR-Egger (β: 0.136, 95% CI −0.420 to 0.692, *p* = 0.632) method also revealed positive correlation between COVID-19 occurrence and risk of bronchitis, they were statistically non-significant (Table 3B, Figure 3B).

**Table 3B:** The MR estimates of the causal effect of COVID-19 on group of diseases associated with airflow blockage and breathing-related problems.

Method	Estimate	SE	95% CI		*p*-value
Simple median	1.077	0.110	0.862	1.292	<0.0001
Weighted median	0.971	0.112	0.751	1.191	<0.0001
Penalized weighted median	1.563	0.109	1.350	1.776	<0.0001
IVW	0.077	0.113	−0.145	0.298	0.497
Penalized IVW	1.638	0.054	1.533	1.743	<0.0001
Robust IVW	0.088	0.132	−0.171	0.347	0.505
Penalized robust IVW	1.771	0.145	1.488	2.055	<0.0001
MR-Egger	0.115	0.248	−0.371	0.600	0.644
Penalized MR-Egger	0.960	0.209	0.550	1.371	<0.0001
Robust MR-Egger	0.136	0.284	−0.420	0.692	0.632
Penalized robust MR-Egger	1.025	0.183	0.667	1.382	<0.0001

### 3.3 Effect of COVID-19 on neurological abnormalities

**Figure 4:**
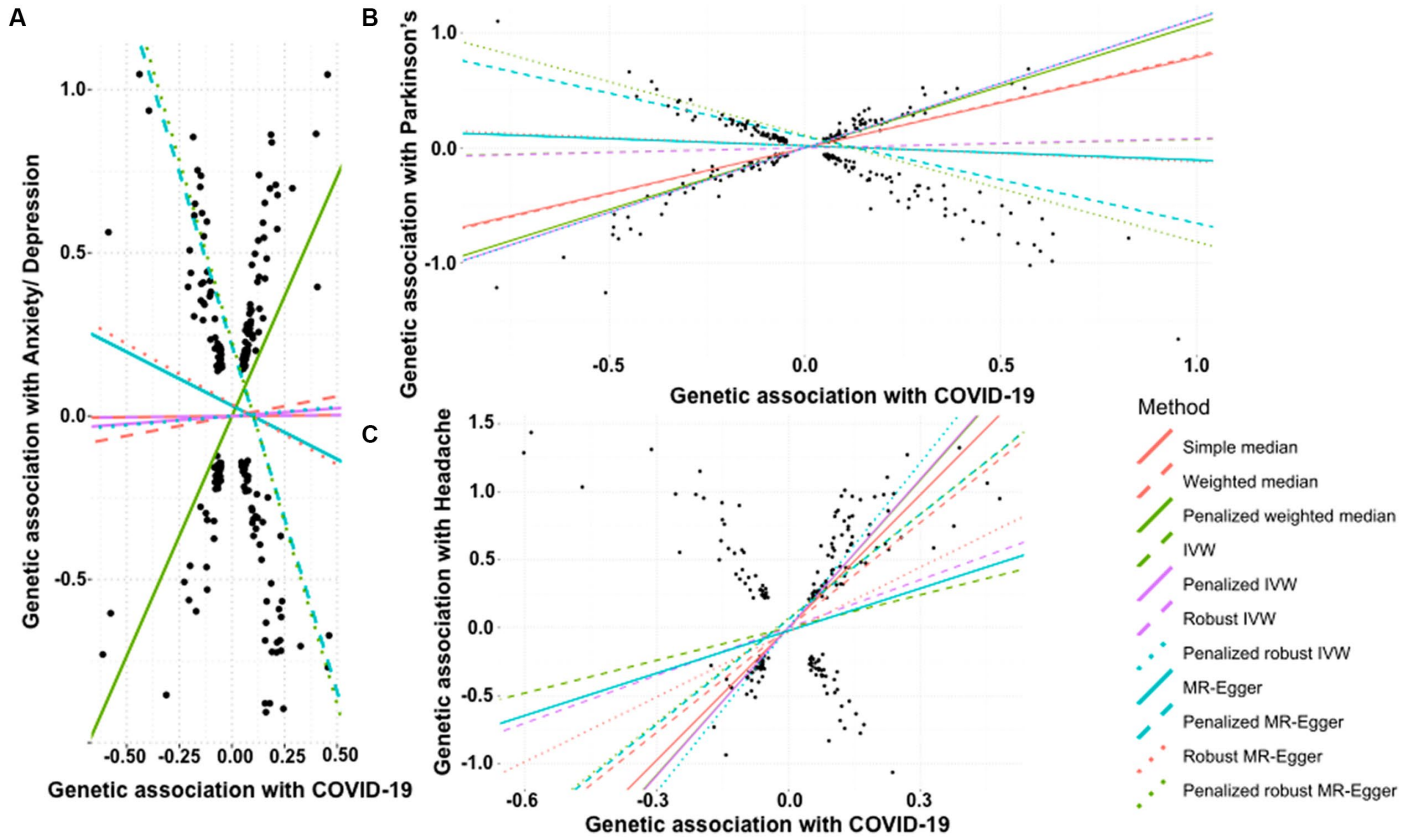
Scatter plot of SNPs associated with COVID-19 and neurological abnormalities. **(A)** anxiety/depression, **(B)** Parkinson’s Disease, and **(C)** headache. The slopes of each line represent the causal association for each method. The plot was generated using the R package MendelianRandomization v0.9.

We did not find any significant association between COVID-19 and the risk of developing anxiety or depression through simple median method (β: 0.007, 95% CI −0.723 to 0.737, *p* = 0.986), weighted median (β: 0.121, 95% CI −0.572 to 0.813, *p* = 0.733), IVW (β: 0.006, 95% CI −0.352 to 0.364, *p* = 0.974), penalized IVW (β: 0.048, 95% CI −0.282 to 0.378, *p* = 0.776), robust IVW method (β: 0.006, 95% CI −0.384 to 0.397, *p* = 0.976), penalized robust IVW (β: 0.055, 95% CI -0.418 to 0.527, *p* = 0.820), MR-Egger (β: −0.328, 95% CI −1.048 to 0.392, *p* = 0.372), and robust MR-Egger (β: −0.370, 95% CI −1.184 to 0.444, *p* = 0.373) (Table 4A). On the contrary, penalized weighted median (β: 1.468, 95% CI 0.892 to 2.044, *p* < 0.0001) indicated a positive association between the two, and penalized MR-Egger (β: −2.121, 95% CI −2.731to −1.510, *p* < 0.0001) and penalized robust MR-Egger (β: −2.220, 95% CI −2.871 to −1.570, *p* < 0.0001) depicted a negative association. Therefore, the association between a history of COVID-19 and risk of anxiety or depression is inconclusive (Table 4A, Figure 4A).

**Table 4A:** The MR estimates of the causal effect of COVID-19 on anxiety/depression.

Method	Estimate	SE	95% CI		*p*-value
Simple median	0.007	0.372	−0.723	0.737	0.986
Weighted median	0.121	0.353	−0.572	0.813	0.733
Penalized weighted median	1.468	0.294	0.892	2.044	<0.0001
IVW	0.006	0.182	−0.352	0.364	0.974
Penalized IVW	0.048	0.168	−0.282	0.378	0.776
Robust IVW	0.006	0.199	−0.384	0.397	0.976
Penalized robust IVW	0.055	0.241	−0.418	0.527	0.820
MR-Egger	−0.328	0.367	−1.048	0.392	0.372
Penalized MR-Egger	−2.121	0.312	−2.731	−1.510	<0.0001
Robust MR-Egger	−0.370	0.415	−1.184	0.444	0.373
Penalized robust MR-Egger	−2.220	0.332	−2.871	−1.570	<0.0001

We tested the effect of COVID-19 on risk of development of Parkinson’s disease. While the simple median (β: 0.785, 95% CI 0.633 to 0.938, *p* < 0.0001) and IVW based methods, i.e., weighted median (β: 0.801, 95% CI 0.697 to 0.905, *p* < 0.0001), penalized weighted median (β: 1.073, 95% CI 0.965 to 1.181, *p* < 0.0001) suggested that the occurrence of COVID-19 is positively associated with an increased risk of Parkinson’s disease. This was also supported by the penalized IVW (β: 1.122, 95% CI 1.066 to 1.177, *p* < 0.0001) and penalized robust IVW (β: 1.124, 95% CI 0.940 to 1.309, *p* < 0.0001) methods. The positive association was also revealed by the IVW (β: 0.075, 95% CI −0.048 to 0.198, *p* = 0.232), robust IVW (β: 0.081, 95% CI −0.063 to 0.225, *p* = 0.270), though non-significant. However, the Egger based methods, MR-Egger (β: −0.124, 95% CI −0.381 to 0.134, *p* = 0.347), penalized MR-Egger (β: −0.753, 95% CI −0.942 to −0.565, *p* < 0.0001), robust MR-Egger method (β: −0.141, 95% CI −0.431 to 0.148, *p* = 0.338) and penalized robust MR-Egger (β: −0.928, 95% CI −1.160 to −0.696, *p* < 0.0001) suggested a negative association between the two. Therefore, the association between a history of COVID-19 and onset of Parkinson’s disease remains undetermined (Table 4B, Figure 4B).

**Table 4B:** The MR estimates of the causal effect of COVID-19 on Parkinson’s disease.

Method	Estimate	SE	95% CI		*p*-value
Simple median	0.785	0.078	0.633	0.938	<0.0001
Weighted median	0.801	0.053	0.697	0.905	<0.0001
Penalized weighted median	1.073	0.055	0.965	1.181	<0.0001
IVW	0.075	0.063	−0.048	0.198	0.232
Penalized IVW	1.122	0.028	1.066	1.177	<0.0001
Robust IVW	0.081	0.073	−0.063	0.225	0.270
Penalized robust IVW	1.124	0.094	0.940	1.309	<0.0001
MR-Egger	−0.124	0.131	−0.381	0.134	0.347
Penalized MR-Egger	−0.753	0.096	−0.942	−0.565	<0.0001
Robust MR-Egger	−0.141	0.148	−0.431	0.148	0.338
Penalized robust MR-Egger	−0.928	0.119	−1.160	−0.696	<0.0001

In contrast to the above, we note a strong positive causal association of a history of COVID-19 with the development of headache by simple median analysis (β: 3.253, 95% CI 2.872 to 3.633, *p* < 0.0001), weighted median (β: 2.572, 95% CI 2.133 to 3.010, *p* < 0.0001), penalized weighted median (β: 3.614, 95% CI 3.129 to 4.098, *p* < 0.0001), IVW (β: 0.802, 95% CI 0.273 to 1.330, *p* = 0.003), penalized IVW (β: 3.637, 95% CI 3.400 to 3.874, *p* < 0.0001), robust IVW (β: 1.171, 95% CI 0.018 to 2.325, *p* = 0.047), penalized robust IVW (β: 4.030, 95% CI 3.283 to 4.778, *p* < 0.0001), penalized MR-Egger (β: 2.578, 95% CI 1.987 to 3.169, *p* < 0.0001) and penalized robust MR-Egger (β: 2.553 95% CI 2.032 to 3.074, *p* < 0.0001) methods. The positive association was also revealed by MR-Egger (β: 1.039, 95% CI −0.093 to 2.170, *p* = 0.072) and robust MR-Egger (β: 1.589, 95% CI −0.196 to 3.375, *p* = 0.081) methods, however they were marginally significant (Table 4C, Figure 4C).

**Table 4C:** The MR estimates of the causal effect of COVID-19 on headache.

Method	Estimate	SE	95% CI		*p*-value
Simple median	3.253	0.194	2.872	3.633	<0.0001
Weighted median	2.572	0.224	2.133	3.010	<0.0001
Penalized weighted median	3.614	0.247	3.129	4.098	<0.0001
IVW	0.802	0.270	0.273	1.330	0.003
Penalized IVW	3.637	0.121	3.400	3.874	<0.0001
Robust IVW	1.171	0.589	0.018	2.325	0.047
Penalized robust IVW	4.030	0.381	3.283	4.778	<0.0001
MR-Egger	1.039	0.577	−0.093	2.170	0.072
Penalized MR-Egger	2.578	0.302	1.987	3.169	<0.0001
Robust MR-Egger	1.589	0.911	−0.196	3.375	0.081
Penalized robust MR-Egger	2.553	0.266	2.032	3.074	<0.0001

### 3.4 Effect of COVID-19 on cardiovascular diseases

**Figure 5:**
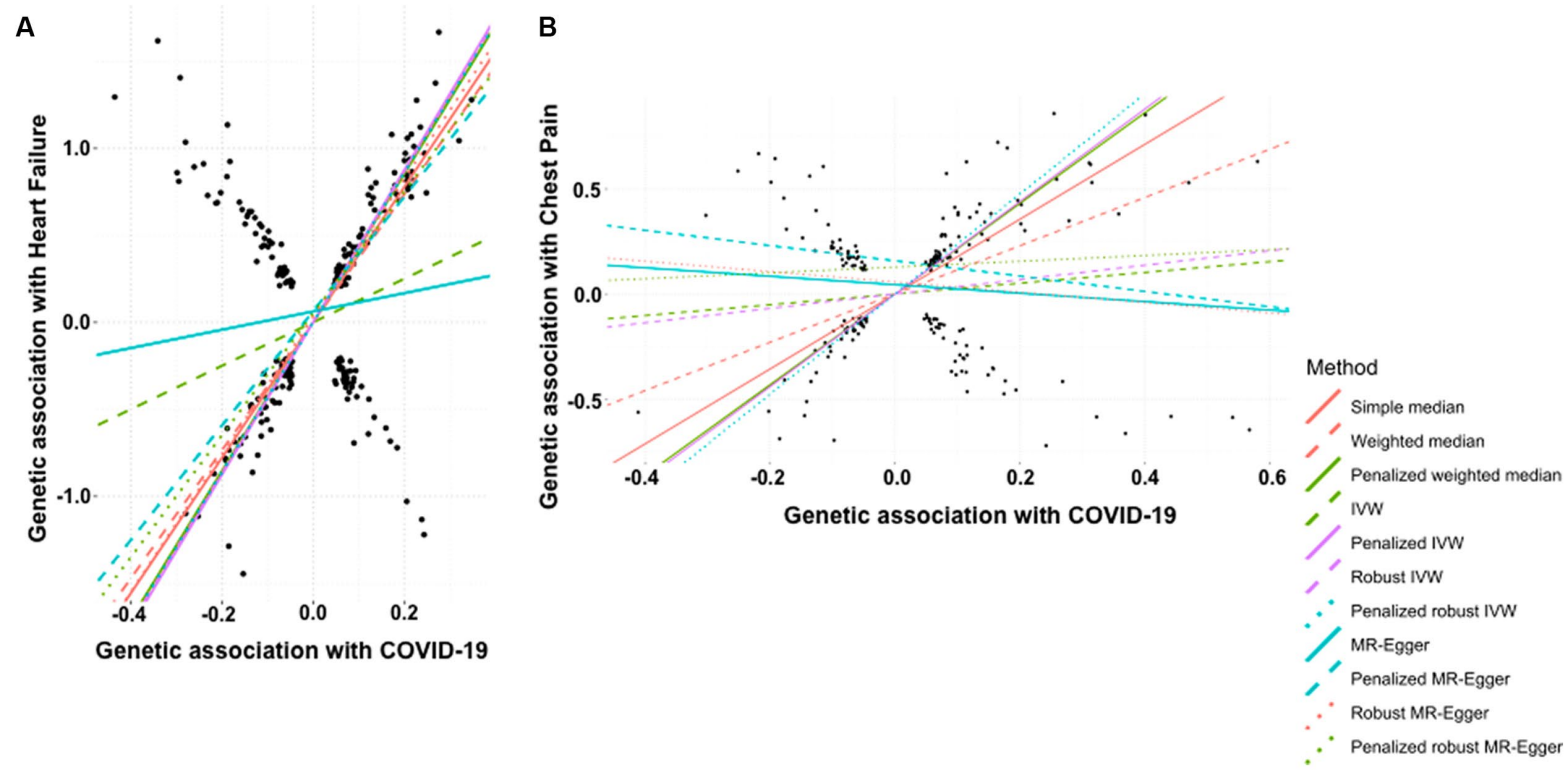
Scatter plot of SNPs associated with COVID-19 and cardiovascular diseases. **(A)** heart failure and **(B)** chest pain. The slopes of each line represent the causal association for each method. The plot was generated using the R package MendelianRandomization v0.9.

The history of COVID-19 showed a strong positive causal association with the risk of heart failure. This was estimated by simple median analysis (β: 0.389, 95% CI 0.355 to 0.423, *p* < 0.0001), weighted median (β: 0.368, 95% CI 0.333 to 0.403, *p* < 0.0001), penalized weighted median (β: 0.427, 95% CI 0.385 to 0.469, *p* < 0.0001), IVW (β: 0.125, 95% CI 0.075 to 0.175, *p* < 0.0001), penalized IVW (β: 0.438, 95% CI 0.416 to 0.460, *p* < 0.0001), robust IVW (β: 0.433, 95% CI 0.407 to 0.460, *p* < 0.0001), penalized robust IVW (β: 0.430, 95% CI 0.358 to 0.502, *p* < 0.0001), penalized MR-Egger (β: 0.329, 95% CI 0.274 to 0.384, *p* < 0.0001), robust MR-Egger (β: 0.396, 95% CI 0.353 to 0.438, *p* < 0.0001) and penalized robust MR-Egger (β: 0.350 95% CI 0.310 to 0.391, *p* < 0.0001) methods. The MR-Egger method also showed a positive association though statistically non-significant (β: 0.053, 95% CI −0.064 to 0.169, *p* = 0.378) (Table 5A, Figure 5A).

**Table 5A:** The MR estimates of the causal effect of COVID-19 on heart failure.

Method	Estimate	SE	95% CI		*p*-value
Simple median	0.389	0.017	0.355	0.423	<0.0001
Weighted median	0.368	0.018	0.333	0.403	<0.0001
Penalized weighted median	0.427	0.021	0.385	0.469	<0.0001
IVW	0.125	0.025	0.075	0.175	<0.0001
Penalized IVW	0.438	0.011	0.416	0.460	<0.0001
Robust IVW	0.433	0.013	0.407	0.460	<0.0001
Penalized robust IVW	0.430	0.037	0.358	0.502	<0.0001
MR-Egger	0.053	0.060	−0.064	0.169	0.378
Penalized MR-Egger	0.329	0.028	0.274	0.384	<0.0001
Robust MR-Egger	0.396	0.022	0.353	0.438	<0.0001
Penalized robust MR-Egger	0.350	0.021	0.310	0.391	<0.0001

According to the simple median analysis, COVID-19 is positively and causatively correlated with the risk of developing chest pain (β: 1.782, 95% CI 1.549 to 2.016, *p* < 0.0001). Similar positive association emerged using weighted median (β: 1.149, 95% CI 0.850 to 1.447, *p* < 0.0001), penalized weighted median (β: 2.158, 95% CI 1.876 to 2.440, *p* < 0.0001), penalized IVW method (β: 2.202, 95% CI 2.062 to 2.343, *p* < 0.0001) and penalized robust IVW (β: 2.382, 95% CI 1.928 to 2.835, *p* < 0.0001) methods. The positive association was further replicated by the IVW (β: 0.256, 95% CI −0.047 to 0.560, *p* = 0.098) and robust IVW (β: 0.343, 95% CI −0.195 to 0.880, *p* = 0.212) methods, however they were statistically non-significant. In contrast all MR-Egger based methods, namely MR-Egger (β: −0.201, 95% CI −0.830 to 0.428, *p* = 0.531), penalized MR-Egger (β: −0.364, 95% CI −0.807 to 0.080, *p* = 0.108) and robust MR-Egger (β: −0.246, 95% CI −1.105 to 0.613, *p* = 0.575) showed negative association between occurrence of COVID-19 and risk of chest pain. Accordingly, the association of COVID-19 with chest pain remains inconclusive (Table 5B, Figure 5B).

**Table 5B:** The MR estimates of the causal effect of COVID-19 on chest pain.

Method	Estimate	SE	95% CI		*p*-value
Simple median	1.782	0.119	1.549	2.016	<0.0001
Weighted median	1.149	0.152	0.850	1.447	<0.0001
Penalized weighted median	2.158	0.144	1.876	2.440	<0.0001
IVW	0.256	0.155	−0.047	0.560	0.098
Penalized IVW	2.202	0.072	2.062	2.343	<0.0001
Robust IVW	0.343	0.274	−0.195	0.880	0.212
Penalized robust IVW	2.382	0.231	1.928	2.835	<0.0001
MR-Egger	−0.201	0.321	−0.830	0.428	0.531
Penalized MR-Egger	−0.364	0.226	−0.807	0.080	0.108
Robust MR-Egger	−0.246	0.438	−1.105	0.613	0.575
Penalized robust MR-Egger	0.138	0.802	−1.433	1.709	0.863

### 3.5 Effect of COVID-19 on chronic fatigue

**Figure 6:**
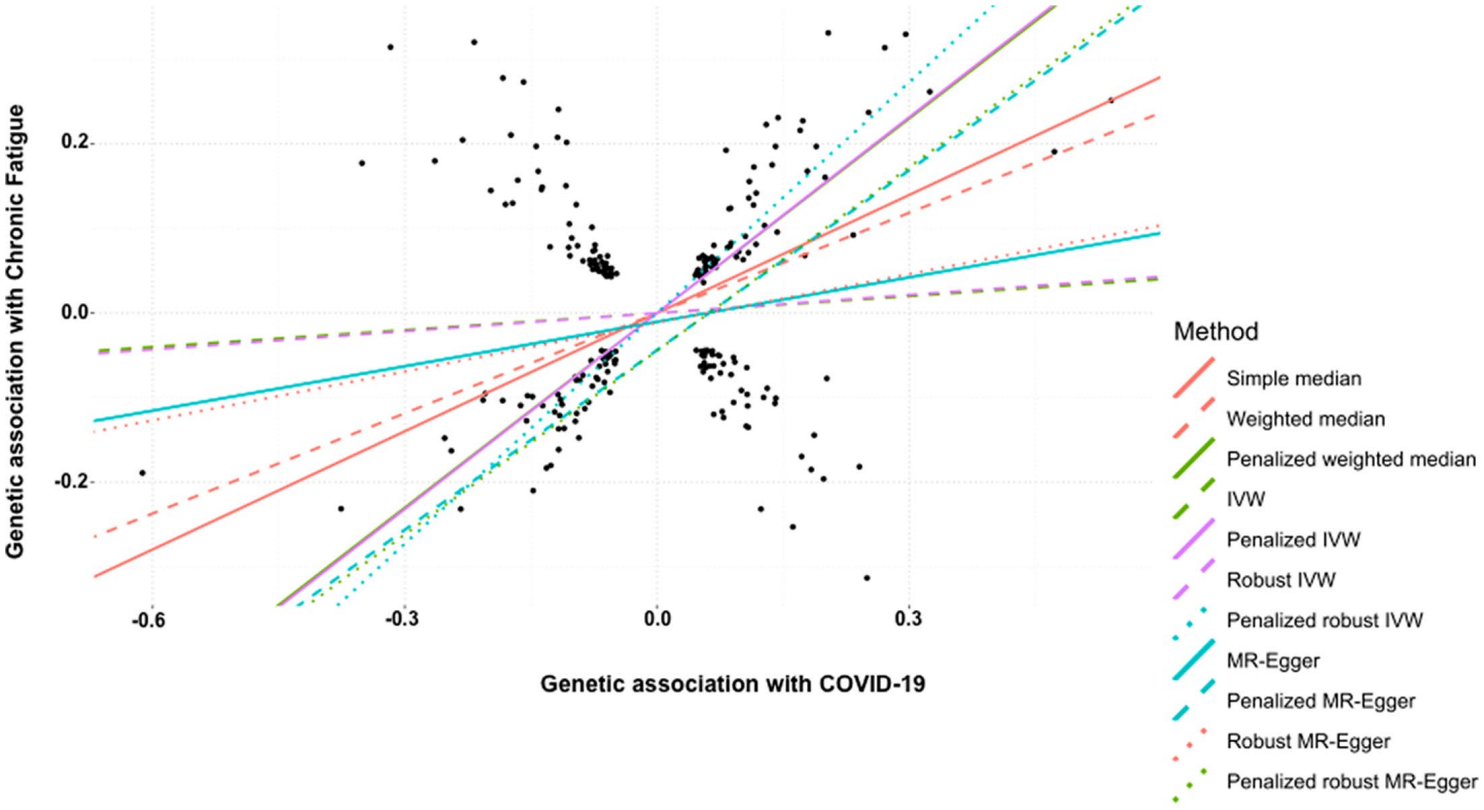
Scatter plot of SNPs associated with COVID-19 and chronic fatigue (tiredness/lethargy). The slopes of each line represent the causal association for each method. The plot was generated using the R package MendelianRandomization v0.9.

Finally, the history of COVID-19 showed a strong and positive association with the risk of developing chronic fatigue (tiredness/lethargy). This was supported by simple median (β: 0.465, 95% CI 0.361 to 0.569, *p* < 0.0001), weighted median (β: 0.394, 95% CI 0.283 to 0.506, *p* < 0.0001), penalized weighted median (β: 0.766, 95% CI 0.652 to 0.879, *p* < 0.0001), penalized IVW (β: 0.771, 95% CI 0.720 to 0.821, *p* < 0.0001), penalized robust IVW (β: 0.908, 95% CI 0.798 to 1.017, *p* < 0.0001), penalized MR-Egger (β: 0.707, 95% CI 0.534 to 0.881, *p* < 0.0001) and penalized robust MR-Egger (β: 0.722, 95% CI 0.525 to 0.920, *p* < 0.0001) methods. IVW (β: 0.067, 95% CI −0.043 to 0.176, *p* = 0.235), robust IVW (β: 0.072 95% CI −0.050 to 0.193, *p* = 0.247), MR-Egger (β: 0.175, 95% CI −0.062 to 0.411, *p* = 0.147) and robust MR-Egger (β: 0.192, 95% CI −0.042 to 0.426, *p* = 0.108) methods also indicted positive association between the two, though statistically non-significant (Table 6, Figure 6).

**Table 6:** The MR estimates of the causal effect of COVID-19 on fatigue (tiredness/lethargy).

Method	Estimate	SE	95% CI		*p*-value
Simple median	0.465	0.053	0.361	0.569	<0.0001
Weighted median	0.394	0.057	0.283	0.506	<0.0001
Penalized weighted median	0.766	0.058	0.652	0.879	<0.0001
IVW	0.067	0.056	−0.043	0.176	0.235
Penalized IVW	0.771	0.026	0.720	0.821	<0.0001
Robust IVW	0.072	0.062	−0.050	0.193	0.247
Penalized robust IVW	0.908	0.056	0.798	1.017	<0.0001
MR-Egger	0.175	0.121	−0.062	0.411	0.147
Penalized MR-Egger	0.707	0.088	0.534	0.881	<0.0001
Robust MR-Egger	0.192	0.119	−0.042	0.426	0.108
Penalized robust MR-Egger	0.722	0.101	0.525	0.920	<0.0001

## 4 Discussion

The COVID-19 pandemic remains an ongoing challenge globally with ~77,04,37,327 confirmed cases worldwide that have not only led to ~69,56,900 deaths (43) (last accessed 11th September, 2023), but has been followed by the emergence of long COVID, which is a complex, multi-systemic entity affecting at least 10% of SARS-CoV-2 infections (2). The heterogeneity of symptoms (≥200) in long COVID patients makes it challenging to dissect which symptoms arise as a cause of SARS-CoV-2 infection vs. those resulting from exacerbation of pre-existing or coincidental conditions. These factors present significant challenges for understanding the pathomechanisms at play and for developing treatment strategies for long COVID. Here we tested the causal association of a genetic predisposition to COVID-19 with several health problems observed among subjects with long COVID, including respiratory, neurological, and cardiovascular dysfunction. Two-sample MR studies in this study suggest that a genetic predisposition for COVID-19 is causally associated with increased risk of fatigue, development of airflow blockage and respiratory problems including bronchitis, emphysema, asthma and rhinitis, pneumonia, headache, and heart failure. However, the association of COVID-19 with Parkinson’s disease, depression and chest pain was inconclusive.

Persistent fatigue is one of the most reported long COVID symptoms worldwide (1, 16). Studies showed that a subset of subjects, ~20% who recover from SARS-COV-2 infections, particularly mild infections, manifest with dyspnea and persistent fatigue despite normal cardiac and pulmonary function (44, 45). Especially in cohorts of long COVID patients with a history of SARS-CoV-2 induced hospitalization chronic fatigue was observed in a higher proportion ~40–60% of affected individuals (46, 47). Myalgic encephalomyelitis/chronic fatigue syndrome (ME/CFS) that is triggered by many pathogens, e.g., Epstein–Barr (EBV) and *Giardia lamblia* in a subset of infections (48, 49) was also noted in a subgroup, ~50% of long COVID subjects (44) including in young individuals with mild or moderate SARS-CoV-2 infections (50). Congruently, MR studies here showed a strong positive association of a genetic predisposition for COVID-19 and persistent fatigue (tiredness/lethargy). While fatigue is likely multisystemic, the pathomechanisms underlying chronic fatigue in long COVID involve neural dysregulation, such as underactivity in particular cortical circuits, abnormal autonomic function, and myopathic changes in skeletal muscle (51). This has also been correlated with structural changes in the thalamus and basal ganglia in long COVID patients with sustained fatigue (52).

A spectrum of neurological symptoms has been reported at varying frequencies in the post-infection phase among long COVID patients. This is consistent with the neurotropic, neuroinvasive and neurovirulent nature of SARS-CoV-2 (53). In some subjects long COVID neuropathogenesis is marked by structural brain anomalies, such as pronounced decrease in its overall size, reduction in gray matter thickness and tissue damage in primary olfactory cortex (54), encephalopathy (55), hemorrhagic posterior reversible encephalopathy (56), and demyelinating lesions in the central nervous system (CNS) (57) in addition to cognitive decline (25). Other factors influencing the neuropathology of long COVID may include neuroinflammation, anti-neural auto-immune dysfunction, hypometabolism of the brain and brain stem, and abnormal cerebrospinal fluid (58–61). Congruently neuroinflammation injury and apoptosis, brain hypoxia and microhaemorrhages have been observed in non-human models of SARS-CoV-2 infection (62). Further neurological and cognitive impairment marked by structural brain abnormalities have also been noted among long COVID subjects following mild or moderate initial SARS-CoV-2 infections (54, 61).

Frequency of chronic headache range widely from ~8–40% among long COVID cases (46, 63–65). Individuals mildly affected by SARS-CoV-2 infections seemed more prone to post-COVID headaches (64, 66), which were exacerbated with a prior history of migraines (63, 64). Our MR analysis showed a strong positive causal association between COVID-19 and chronic headache in long COVID patients. Recent evidence suggests that long COVID headaches appear to be triggered by hyperinflammation and are sustained by chronic inflammatory activation, and dysregulation of neurotransmitters and metabolic inflammation (66).

Based on observational studies, among individuals affected with long COVID, ~20% may develop mood disorders, e.g., anxiety and depression (47). In one study the risk of mood disorders returned to baseline within 2 months following initial COVID-19, but some conditions such as cognitive impairment, seizures, psychosis, and dementia lingered up to 2 years (25). Another study noted that limbic atrophy and significantly abnormal cerebral functional connectivity underlie anxiety and depression among long COVID patients with mild SARS-CoV-2 infections (67). In contrast several studies inferred that anxiety and depression were not strongly linked to COVID-19 (68) with neuropsychiatric symptoms elevated disproportionately in long COVID subjects with acute initial SARS-CoV-2 infection (68, 69) or other post-infection health complications and psychiatric history (70). The onset and progression of mood disorders may also be modulated by dysfunctional regulatory cells of the innate and adaptive immune system that may contribute to chronic systemic and neuroinflammation (71). Therefore, the observations of increased psychiatric manifestations in long COVID subjects could be a result of compromised immunoregulatory mechanisms. Using MR in the present study the causal association of genetic predisposition to COVID-19 with anxiety and depression remained obscure, warranting further research to clarify this.

Parkinson’s disease is a progressive motor disorder that is highly prevalent in older adults (72). It may be triggered following infections by viruses, e.g., Influenza A, EBV and Herpes simplex virus 1 (73). Some cases of post-COVID parkinsonism have also been recorded (26, 27). Nevertheless, we detected no causal association of COVID-19 with Parkinson’s disease using MR studies.

A range of cardiovascular manifestations have been noted in long COVID patients, including those without any evidence of pre-existing cardiovascular disease or risk factors or COVID-19 related hospitalization (29). While the pathophysiology of long COVID linked cardiovascular disease is far from certain, it may involve viral invasion of cardiomyocytes and cell death, downregulation of Angiotensin-converting enzyme 2 (ACE2), endothelial cell infection, complement activation, deregulation of renin-angiotensin-aldosterone system, autonomic abnormalities, myocarditis and cardiac tissue fibrosis (74–79). The proportion of long COVID subjects with chest pain varied from ~3–20% up to 6 months post initial SARS-CoV-2 infection in different cohorts (80, 81). While the overall association of genetic predisposition to COVID-19 with chest pain in this study was inconclusive, five out of twelve MR strategies showed a strong positive causal link that was statistically significant, two techniques showed a positive association that was non-significant, and the rest showed negative association. This strongly warrants further studies to explore a putative causal link of COVID-19 with chest pain.

Heart failures as part of cardiovascular sequalae in long COVID have been observed in patients with pre-existing cardiac complications (82, 83), as well as those without (84–86). This is also supported by anecdotal accounts of increased cardiovascular abnormalities particularly among mildly symptomatic or asymptomatic subjects in the post SARS-CoV-2 infection period. Studies note that autonomic imbalance and increased sympathetic activity in the post-acute phase in ~30 days after mild SARS-CoV-2 infection may explain the cardiovascular complications during this time (87, 88). Although, the involvement of autonomic dysfunction in heart complications in long COVID is uncertain (87). All 11 methods employed for MR analysis in this study suggested a positive causal association between genetic predisposition to COVID-19 and heart failures. Ten out of eleven methods concur on this with high significance (*p* < 0.0001). Moreover, the effect size (β) range is highly consistent across all methods with narrow confidence intervals. Taken together MR analysis suggested a strong and positive causal association between genetic predisposition to COVID-19 and heart failure.

Finally, respiratory complications, e.g., cough are one of the commonest features of long COVID subjects (4) and are exacerbated in patients with preexisting respiratory comorbidities (89). Subjects with moderate-to-severe SARS-CoV-2 related pneumonia sustain lung abnormalities, such as parenchymal lung disease, fibrosis, and bronchiectasis even a year following initial infection (90). New onset of airway abnormalities, e.g., asthma is not common but has been strongly associated with COVID-19 (91). Increased lung emphysema was noted at 30 days following initial SARS-CoV-2 infection (92). In this study MR results support a strong positive causal association of a history of COVID-19 with increased risk of disorders with chronic lung and airway inflammation, as well as pneumonia.

This study has some limitations. First since it utilizes publicly available genetic datasets, the vaccination and BTI status of the included individuals is unknown. Accordingly, it cannot account for how these factors influence long COVID outcomes. It is noteworthy that BTI of SARS-CoV-2 following vaccination may be influenced by several factors, e.g., age and previous infection (93). Moreover, the serological response to vaccines was also reported to be insufficient in individuals with two or more pre-existing chronic conditions (94). These factors may influence long COVID phenotypes and cannot be evaluated in the present study. Second, the dataset for chest pain used here is non-specific, and may include subjects where it is caused due to cardiac, lung or abdominal organ abnormalities. This may also explain why the association of a history of COVID-19 with chest pain in this study was inconclusive despite it being a commonly noted long COVID complexity (2).

In conclusion, this study has employed MR to shed light on the genetic underpinnings of long COVID, uncovering significant causal associations between genetic predisposition to COVID-19 and several prevailing health complications. MR findings in this study corroborate the multiple adverse outcomes of long COVID, linking it to an increased risk of developing pneumonia, airway infections, headache, chronic fatigue, and heart failure. Our findings on Parkinson’s disease, depression, and chest pain were inconclusive. These insights are critical in enhancing our understanding of the lasting health implications of COVID-19. As we continue to grapple with the long-term challenges posed by the COVID-19 pandemic, a deeper comprehension of the genetic factors contributing to long COVID will be a precursor to developing targeted strategies to support those symptomatic with these long COVID symptoms. Further research is warranted to explore the intricate web of genetic determinants underlying the diverse manifestations of long COVID, which will spearhead global efforts in combating this healthcare crisis effectively.

## Data availability statement

Publicly available datasets were analyzed in this study. This data can be found here: https://gwas.mrcieu.ac.uk/ and https://www.covid19hg.org/results/r6/.

## Ethics statement

Ethical approval was not required for the study involving humans in accordance with the local legislation and institutional requirements. Written informed consent to participate in this study was not required from the participants or the participants’ legal guardians/next of kin in accordance with the national legislation and the institutional requirements.

## Author contributions

PS: Methodology, Visualization, Writing – review & editing, Data curation, Formal Analysis, Software. HU: Writing – review & editing, Funding acquisition, Investigation, Project administration, Resources. JKS: Writing – review & editing, Data curation, Formal Analysis, Software. SB: Data curation, Formal Analysis, Software, Writing – review & editing. NJ: Data curation, Formal Analysis, Software, Writing – review & editing. NK: Data curation, Formal Analysis, Software, Writing – review & editing. RD: Conceptualization, Data curation, Formal Analysis, Investigation, Methodology, Software, Supervision, Validation, Visualization, Writing – review & editing. PU: Writing – review & editing, Conceptualization, Investigation, Methodology, Validation, Visualization, Writing – original draft.

## Footnotes

^1^ https://gwas.mrcieu.ac.uk/datasets
