# Erratum: The impact of COVID-19 on pulmonary, neurological, and cardiac outcomes: evidence from a Mendelian randomization study

**DOI:** 10.3389/fpubh.2024.1380811

**Published:** 2024-02-12

**Authors:** Frontiers Production Office

**Affiliations:** Frontiers Media SA, Lausanne, Switzerland

**Keywords:** long COVID, Mendelian randomization, COVID-induced neurological disorders, COVID-induced fatigue, COVID-induced cardiac disease

Due to a production error, the reference “93” was incorrectly written as “Porru S et al. Post-Vaccination SARS-CoV-2 Infections among Health Workers at the University Hospital of Verona, Italy: A Retrospective Cohort Survey. Vaccines (Basel). 2022 Feb 10;10(2):272. doi: 10.3390/vaccines10020272”. This error resulted in an erroneous Scopus indexation. It should be “Porru, S, Monaco, MGL, Spiteri, G, Carta, A, Pezzani, MD, Lippi, G, et al. SARS-CoV-2 breakthrough infections: incidence and risk factors in a large European multicentric cohort of health workers. Vaccines (Basel). (2022) 10:1193. doi: 10.3390/vaccines10020272”.

The publisher apologizes for this mistake.

The original article has been updated.

