# Supplementary material for: The impact of COVID-19 on pulmonary, neurological, and cardiac outcomes: evidence from a Mendelian randomization study
Source: Front Public Health. 2023 Dec 14;11:1303183. doi: 10.3389/fpubh.2023.1303183 (PMC10752946; doi:10.3389/fpubh.2023.1303183)
Supplement: Supplementary file 1 [file Data_Sheet_1.pdf]

Supplementary Material

1.1 Supplementary Figures

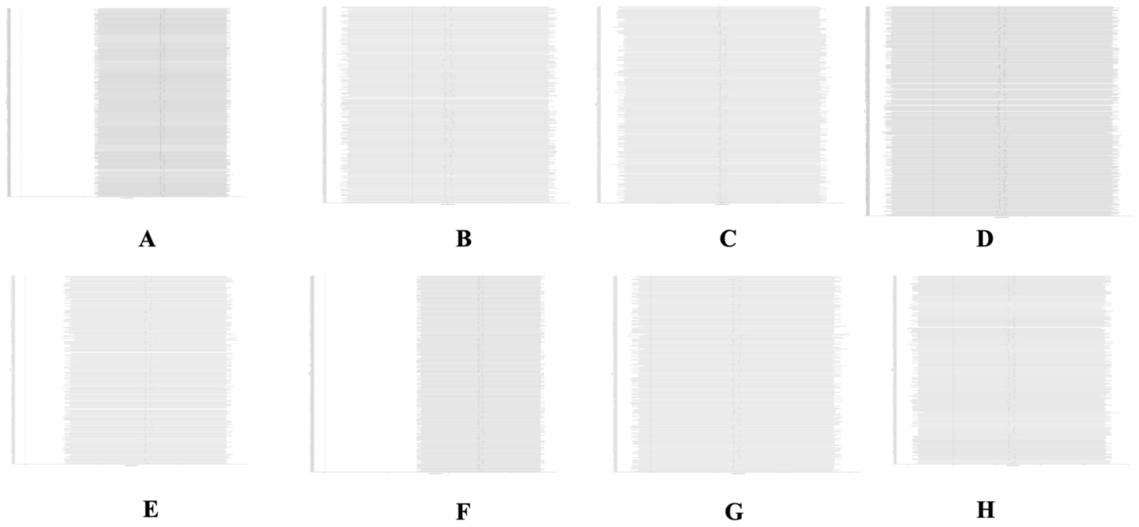

Supplementary Figure 1. "leave-one-out" sensitivity analysis, performed using Mendelian Randomization v0.9 for the effect of COVID-19 on A. pneumonia, B. airflow blockage and breathing-related problems such as bronchitis, C. anxiety/depression, D. Parkinson's Disease, E. headache, F. heart failure, G. chest pain and H. chronic fatigue (tiredness/lethargy). The x-axis shows the leave-one-out sensitivity analysis for COVID-19 on these disorders. The y-axis shows the analysis for the effect of leave-one-out of SNPs on these disorders. The leave-one-out sensitivity analysis indicated that removing a specific SNP among the 32,756 COVID-19 SNPs did not change the results.

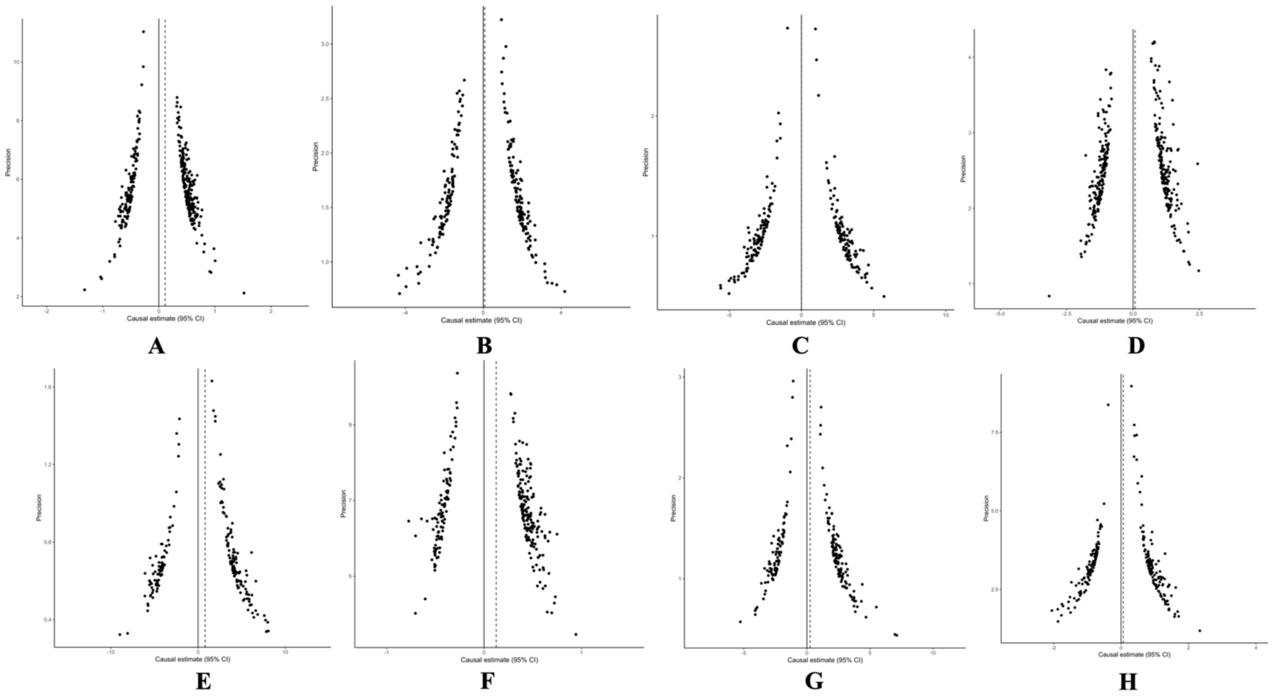

Supplementary Figure 2: Funnel plots of SNPs associated with COVID-19 and A. pneumonia, B. airflow blockage and breathing-related problems such as bronchitis, C. anxiety/depression, D. Parkinson's Disease, E. headache, F. heart failure, G. chest pain and H. chronic fatigue (tiredness/lethargy). The plots were generated using Mendelian Randomization v0.9. The y-axis represents a measure of study precision, and the x-axis displays the study estimated effect size for these disorders. The plot looks like a symmetrical inverted funnel, indicating apparent absence of both bias and heterogeneity.
